# White matter hyperintensities are seen only in *GRN* mutation carriers in the GENFI cohort

**DOI:** 10.1016/j.nicl.2017.04.015

**Published:** 2017-04-26

**Authors:** Carole H. Sudre, Martina Bocchetta, David Cash, David L. Thomas, Ione Woollacott, Katrina M. Dick, John van Swieten, Barbara Borroni, Daniela Galimberti, Mario Masellis, Maria Carmela Tartaglia, James B. Rowe, Caroline Graff, Fabrizio Tagliavini, Giovanni Frisoni, Robert Laforce, Elizabeth Finger, Alexandre de Mendonça, Sandro Sorbi, Sébastien Ourselin, M. Jorge Cardoso, Jonathan D. Rohrer, Genetic FTD Initiative, GENFI

**Affiliations:** a Dementia Research Centre, Department of Neurodegenerative Disease, UCL Institute of Neurology, Queen Square, London, UK; b Centre for Medical Image Computing, University College London, UK; c Erasmus Medical Center, Rotterdam, Netherlands; d University of Brescia, Italy; e Dept. of Pathophysiology and Transplantation, “Dino Ferrari” Center, University of Milan, Fondazione Cà Granda, IRCCS Ospedale Maggiore Policlinico, Milan, Italy; f Cognitive Neurology Research Unit, Sunnybrook Health Sciences Centre, Hurvitz Brain Sciences Research Program, Sunnybrook Research Institute; Department of Medicine, University of Toronto, Canada; g Tanz Centre for Research in Neurodegenerative Diseases, University of Toronto, Canada; h University of Cambridge, UK; i Karolinska Institutet, Stockholm, Sweden; j Karolinska Institutet, Department NVS, Center for Alzheimer Research, Division of Neurogeriatrics, Sweden; k Department of Geriatric Medicine, Karolinska University Hospital, Stockholm, Sweden; l Istituto Neurologico Carlo Besta, Milan, Italy; m IRCCS San Giovanni di Dio Fatebenefratelli Brescia, Italy; n Université Laval, Quebec, Canada; o University of Western Ontario, Ontario, Canada; p Faculdade de Medicina, Universidade de Lisboa, Portugal; q Department of Neurosciences, Psychology, Drug Research and Child Health (NEUROFARBA), University of Florence, Florence, Italy; r IRCCS Don Gnocchi, Firenze, Italy

**Keywords:** PS, Presymptomatic, S, Symptomatic, FTD, Frontotemporal dementia, WMH, White matter hyperintensity, IQR, Inter Quartile Range, CI, Confidence interval, TIV, Total Intracranial volume

## Abstract

Genetic frontotemporal dementia is most commonly caused by mutations in the progranulin *(GRN)*, microtubule-associated protein tau (*MAPT)* and chromosome 9 open reading frame 72 *(C9orf72*) genes. Previous small studies have reported the presence of cerebral white matter hyperintensities (WMH) in genetic FTD but this has not been systematically studied across the different mutations. In this study WMH were assessed in 180 participants from the Genetic FTD Initiative (GENFI) with 3D T1- and T2-weighed magnetic resonance images: 43 symptomatic (7 *GRN*, 13 *MAPT* and 23 *C9orf72*), 61 presymptomatic mutation carriers (25 *GRN*, 8 *MAPT* and 28 *C9orf72*) and 76 mutation negative non-carrier family members. An automatic detection and quantification algorithm was developed for determining load, location and appearance of WMH. Significant differences were seen only in the symptomatic *GRN* group compared with the other groups with no differences in the *MAPT* or *C9orf72* groups: increased global load of WMH was seen, with WMH located in the frontal and occipital lobes more so than the parietal lobes, and nearer to the ventricles rather than juxtacortical. Although no differences were seen in the presymptomatic group as a whole, in the *GRN* cohort only there was an association of increased WMH volume with expected years from symptom onset. The appearance of the WMH was also different in the *GRN* group compared with the other groups, with the lesions in the *GRN* group being more similar to each other. The presence of WMH in those with progranulin deficiency may be related to the known role of progranulin in neuroinflammation, although other roles are also proposed including an effect on blood-brain barrier permeability and the cerebral vasculature. Future studies will be useful to investigate the longitudinal evolution of WMH and their potential use as a biomarker as well as post-mortem studies investigating the histopathological nature of the lesions.

## Highlights

• In genetic FTD white matter hyperintensities (WMH) are found most prominently in symptomatic patients with GRN mutations.
• Frontal and occipital lobes are the most affected regions.
• WMH are more likely to occur close to the ventricles.
• WMH have a more homogenous appearance possibly suggestive of inflammatory rather than vascular lesions.

## 1 Introduction

Frontotemporal dementia (FTD) is an umbrella term used to denote a group of neurodegenerative disorders affecting principally the frontal and temporal lobes. It is a highly heritable disorder with approximately a third of cases being caused by mutations in predominantly three genes: progranulin (*GRN*), microtubule associated protein tau (*MAPT*) and chromosome 9 open reading frame 72 (*C9orf72*) (Rohrer et al., 2015, Rohrer and Warren, 2011). Whilst the clinical features of *GRN-, MAPT*- and *C9orf72*-associated FTD largely overlap, the underlying molecular processes leading to that phenotypic endpoint are fundamentally different (Lashley et al., 2015).

To date, most antemortem studies of familial FTD have focused on changes in gray matter, but some types of FTD are known to be associated with white matter pathology. Such changes may be seen by magnetic resonance imaging (MRI) e.g. cerebral white matter hyperintensities (WMH). These WMH are usually identified on T2, FLAIR or PD-weighted MRI and reflect an abnormal tissue fat/water ratio. They are commonly seen in healthy aging and more extensively in patients with neuroinflammatory disorders or small vessel cerebrovascular disease. Research into the link between WMH and other forms of dementia has been ongoing for some time with lesions seen particularly in those with vascular dementia as well as being commonly associated with Alzheimer's disease (Snyder et al., 2015). In such cases of dementia lesions are generally felt to represent ischaemic damage (Wardlaw et al., 2015). WMH are less commonly seen in FTD but recent small studies have reported their presence in some cases, particularly in those with *GRN* mutations (Caroppo et al., 2014, Paternicò et al., 2016, Pietroboni et al., 2011). However, a detailed investigation in a large cohort has yet to be performed.

Many studies of WMH in dementia use visual rating scales, manual or semi-automated segmentation methods (Yoshita et al., 2005). However, for large cohorts time consuming operator-dependent segmentation becomes unfeasible. Automated segmentation methods have therefore been developed for extracting WMH from either FLAIR or T2-weighted images. We have previously developed a methodology for automatically segmenting WMH through modelling of unexpected observations in MR images (Sudre et al., 2015). In order to investigate the presence of WMH in FTD further we used this methodology on data from the Genetic FTD Initiative (GENFI) (Rohrer et al., 2015) which investigates symptomatic and at-risk members of families with mutations in *GRN, MAPT* and *C9orf72*.

## 2 Methods

### 2.1 Demographics

The first phase of the GENFI multicentre cohort study comprised 13 research centres across Europe and Canada (www.genfi.org.uk) (Rohrer et al., 2015). Local ethics committees gave approval for the study at each site and all participants gave written informed consent prior to enrolment. Between January 2012 and April 2015 365 participants were recruited into GENFI, of whom 190 underwent both 3D T1 and 3D T2 acquisitions on a 3T MRI scanner. Ten scans did not pass quality control (excessive motion) and so 180 were used for the final analysis. Four scanner types were used with protocols designed at the outset of the study to minimise discrepancies between scanners. Of the 180 participants included in the study, 43 were symptomatic (7 *GRN*, 13 *MAPT* and 23 *C9orf72*) and 61 were presymptomatic mutation carriers (25 *GRN*, 8 *MAPT* and 28 *C9orf72*) with a further 76 participants found to be mutation negative non-carriers (and therefore acting as a control group). Demographics for the cohort are described in Table 1.

**Table 1:** Demographics of the cohort and summary of WMH volumes for each group.

		NC	*C9orf72*		*GRN*		*MAPT*	
			PS	S	PS	S	PS	S
	Number Male:female	76 (51:25)	28 (16:12)	23 (5:18)	24 (14:10)	8 (4:4)	8 (7:1)	13 (4:9)
	Mean (SD) age *(years)*	48.2 (15.1)	44.4 (11.3)	66.1 (6.7)	45.7 (12.3)	65.7 (6.6)	38.0 (11.5)	57.9 (8.2)
	Mean (SD) TIV *(ml)*	1523 (139)	1582 (164)	1634 (135)	1535 (128)	1512 (127)	1485 (91)	1555 (120)
WMH *(ml)*	Mean	0.80	0.81	0.93	0.76	2.63	0.43	1.32
	Median	0.49	0.63	0.72	0.46	0.75	0.41	0.60
	Range	[0.04 6.56]	[0.08 2.59]	[0.16 2.46]	[0.11 4.30]	[0.63 7.45]	[0.14 0.87]	[0.02 5.02]
	IQR	[0.32 0.70]	[0.24 1.24]	[0.23 1.50]	[0.23 0.71]	[0.65 5.08]	[0.18 0.64]	[0.34 1.09]
	SD	1.17	0.70	0.71	0.94	2.81	0.27	1.69

NC - Non-carriers; PS - Presymptomatic; S - Symptomatic; SD - Standard deviation; TIV - Total intracranial volume; IQR - Interquartile range.

### 2.2 Imaging analysis

Whilst FLAIR images have become the standard for the study of WMH due to good separation between CSF and lesion signal, T2-weighted images may also be used, although are more challenging to segment due to the proximity in signal signature between lesions and CSF. In order to segment the WMH in the GENFI dataset (which includes 3D T2-weighted images but not FLAIR) our previously described algorithm (Sudre et al., 2015) was adapted to address the specific challenges that arise in the use of T2-images. In this model an adaptive hierarchical three-level multivariate Gaussian mixture model (GMM) using both T1 and T2 weighted images is used to model both normal and unexpected signal observations. At a first level, inliers (I) and outliers (O) are segmented and a priori information on their location is progressively introduced by smoothed maps of typicality measures (Van Leemput et al., 2001). At the second level, anatomical information is introduced through statistical atlases so as to model the different biological tissues (gray matter, white matter, CSF and other non-brain structures) for both the inlier and outlier parts of the model. The appropriate number of Gaussian components necessary to model the different tissues and their parameters are presented at the third level of the hierarchy. In this study, the anatomical atlases are obtained as a result of a label fusion algorithm (Cardoso et al., 2015): Gaussian components parameters are optimised via an expectation-maximisation (EM) algorithm that incorporates contextual constraints with the application of a Markov Random Field. In order to determine the number of Gaussian components required to model the data, splitting and merging operations are tested at the third level of the hierarchy. To ensure a balance between model accuracy and fit, the Bayesian Information Criterion is used to assess if a model change should be accepted or not. The list of model changes to test is determined each time the model complexity evolves and the algorithm stops once all of these changes have been tested and rejected. Once the data model has been determined and optimised, it can be used to segment WMH. The characteristics of the healthy appearing WM are used as a reference to select the voxels classified as outliers that could be considered as lesion. More specifically, the probabilistic maps of outliers are multiplied voxelwise by a weight w_n_ defined as:

**Figure:**
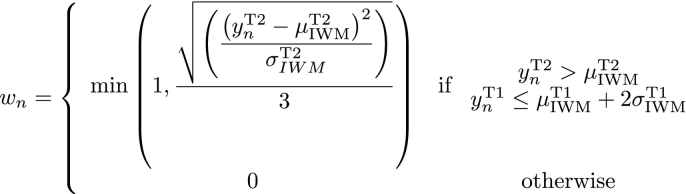

where *y*_*n*_ indicates the intensity at voxel n, and *μ*_IWM_ and *σ*_IWM_refer to the mean and standard deviation of the white matter inliers (IWM). The anatomical tissue segmentation result is then combined with a brain parcellation to expunge the probabilistic map of voxels whose intensity reflect partial volume effect between the main tissues and the ventricular lining. In particular, the ventricular segmentation is morphologically dilated and voxels classified as lesion removed from this area.

**Fig. 1:**
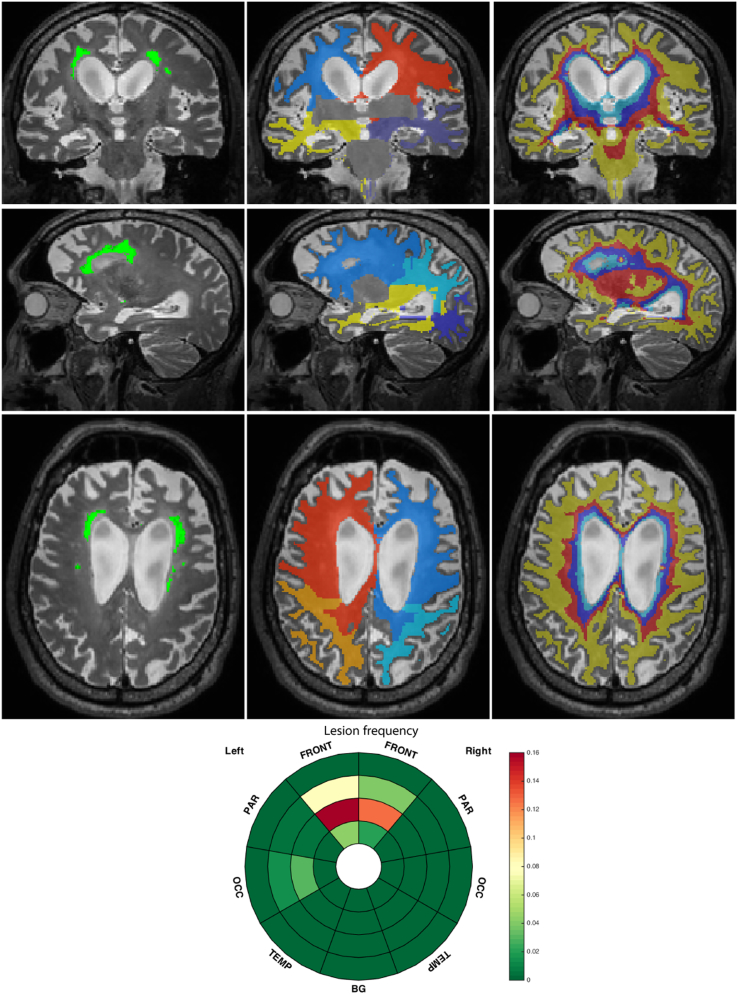
Example of the location patient-specific scheme for the three orientations. From left to right: 1st column: T2-weighted image with overlayed lesion segmentation; 2nd column: lobar separation; 3rd column: layer discretisation. The corresponding lesion frequency per zone is given in the inset image at the bottom.

In determining WMH location, biases may be introduced if non-linear registrations are applied to images with lesions (Chard et al., 2010) or if absolute distance to the ventricular lining that does not account for atrophy is chosen to differentiate between lesion locations (Woong Kim et al., 2008). Additionally, in the case where few lesions are present, voxelwise analyses may prove prone to noise (van der Lijn et al., 2012). Therefore, to study the lesion distribution in the brain, a patient-specific location scheme was applied dividing the white matter into regions reflecting their distance to the ventricular surface and lobes. To separate lobar regions, a parcellation of the gray matter was used to divide it into frontal, parietal, occipital and temporal lobes for the left and the right hemisphere. Euclidean distance from these defined lobes was then used to separate the WM. Basal ganglia were considered as a separate region and the infratentorial region excluded from the analysis. In order to avoid using absolute values that would be biased by brain atrophy, normalised distance maps between ventricular surface and cortical sheet were computed using the solution to the Laplace equation as described by Yezzi et al. (Yezzi and Prince, 2002) and discretised into four equidistant layers as suggested by Kim et al. (Kim et al., 2008) with layer 1 being nearest to the ventricle and layer 4 being juxtacortical. Ultimately the white matter domain was separated into 36 zones (made up of 9 regions and 4 layers within each region). To visualise the zonal separation, a bullseye plot was used in which the regions were encoded by the angular position and the layers were given by the radial position, with the distance to the ventricular surface increasing with the distance from the plot centre. The zonal characteristics such as the proportion of the zone affected by WMH can then be colour-encoded in the plot. With this representation, complex 3D information was thus summarized and gathered into a planar systematic infographic. Fig. 1 presents an illustration of the zonal separation and the lesion segmentation for a case of a symptomatic subject with a *GRN* mutation.

### 2.3 Statistical analyses

Stata v14 was used for all statistical analyses. Due to the skewness of the data, both global and zonal volumetric WMH values were log-transformed before analysis.

For the global volumes analysis, linear regression was performed considering WMH volume as the dependent variable and adjusting for age, gender, total intracranial volume (TIV), years from expected symptom onset [see Rohrer et al., 2015 for more details] and scanner type. A different adjustment was allowed for their mutation status [presymptomatic vs symptomatic] and genetic group [*GRN* vs *C9orf72* vs *MAPT*]. A similar analysis was also performed for the different lobes and layers.

In order to exclude any confounding influence of cardiovascular risk factors, the effects of hypertension, hypercholesterolaemia and diabetes mellitus on WMH volumes were assessed separately for each mutation group. None showed any significant association and were therefore not further included as covariates in the model.

For the location analysis, the zonal standardised log-volumes were used as dependent variables and corrected separately for age, gender, TIV, scanner type and again a different adjustment was allowed for mutation status and genetic group.

A further analysis was performed on the distribution of lesion intensities, which (due to the adopted model for lesion segmentation) can be expressed as a standardised intensity or Z-score to the normal appearing WM tissue and allows groups to be distinguished. Although T2-weighted MR acquisition does not provide a quantitative measurement of the damage to the WM, such an intensity analysis provides an estimate of the appearance of the lesions i.e. how similar the hyperintensities of different lesions are. In order to avoid including participants that present with enlarged perivascular spaces as signal hyperintensities in the WM only participants with at least 0.5 mL of WMH were selected for this analysis.

## 3 Results

### 3.1 Global volumes analysis

WMH volumes are shown in Table 1. Age was significantly associated with WMH volumes (*p* = 0.0005) as was TIV (*p* = 0.003). Mean adjusted back-transformed results with confidence intervals and *p*-values for group comparisons are shown in Table 2: symptomatic *GRN* subjects had a significantly higher mean global WMH volume than presymptomatic *GRN* cases and noncarriers, as well as more than the symptomatic *MAPT* and *C9orf72* groups. By contrast, no significant difference was observed between the presymptomatic *GRN* cases and the noncarriers. Furthermore, no significant differences were seen between the symptomatic (or presymptomatic) *C9orf72* or *MAPT* groups and noncarriers.

**Table 2:** Mean adjusted WMH values corrected for age, sex, TIV, scanner type and estimated average years before onset. Volumetric values are given in m*l*.

Non-carriers		Carriers	Presymptomatic		Symptomatic		*p*-Values
Mean	CI		Mean	CI	Mean	CI	
0.50	[0.40 0.62]	***C9orf72***	0.50	[0.35 0.71]	0.44	[0.28 0.67]	NC vs PS 0.99 NC vs S 0.60 PS vs S 0.87
		***GRN***	0.56	[0.39 0.81]	1.51	[0.77 2.94]	NC vs PS 0.60 NC vs S 0.0030 PS vs S 0.012
		***MAPT***	0.53	[0.27 1.06]	0.59	[0.34 1.02]	NC vs PS 0.87 NC vs S 0.60 PS vs S 0.87
		***p*-values**	*C9orf72* vs *GRN* 0.86 *C9orf72* vs *MAPT* 0.99 *GRN* vs *MAPT* 0.86		*C9orf72* vs *GRN* 0.0048 *C9orf72* vs *MAPT* 0.67 *GRN* vs *MAPT* 0.012		

CI - Confidence interval.

**Fig. 2:**
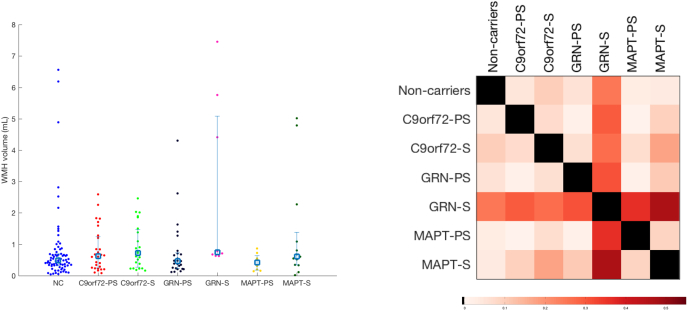
Beeswarm plot of WMH volumes for the different groups (left) and corresponding matrix of effect size of pairwise comparisons.

For visualisation purposes, the beeswarm plot of the WMH volumes for the different groups is presented in Fig. 2 along with a colour-coded representation of the effect size when comparing the different groups.

The presymptomatic groups include a heterogeneous population including some participants near to the mean age of onset in the family and others more distant. In order to evaluate the change in WMH with disease progression, disjoint regression models were used to analyse the relationship between WMH volumes and years from expected age of onset in each mutation group, correcting for gender, scanner type and TIV. An association between WMH volume and years from expected age of onset was only significant in the *GRN* group (*p* = 0.002) [*MAPT* group (*p* = 0.286), *C9orf72* group (*p* = 0.214)].

### 3.2 Location analysis

**Fig. 3:**
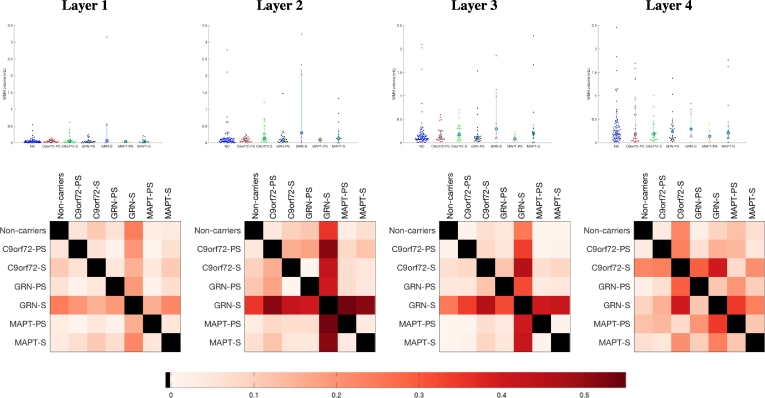
Comparison between groups of the WMH load per layer (Layer 1 being nearest the ventricle, Layer 4 being juxtacortical). The first row presents the beeswarm plots of WMH volumes and the second row the corresponding matrices of effect size in the group comparison.

**Fig. 4:**
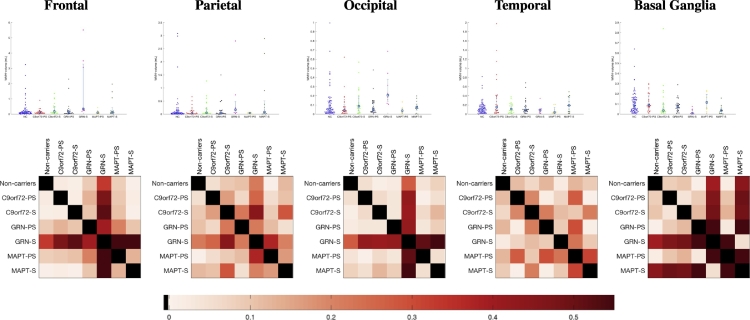
Comparison between groups of the WMH load per lobe. The first row presents the beeswarm plots of WMH volumes and the second row the corresponding matrices of effect size in the group comparison.

**Fig. 5:**
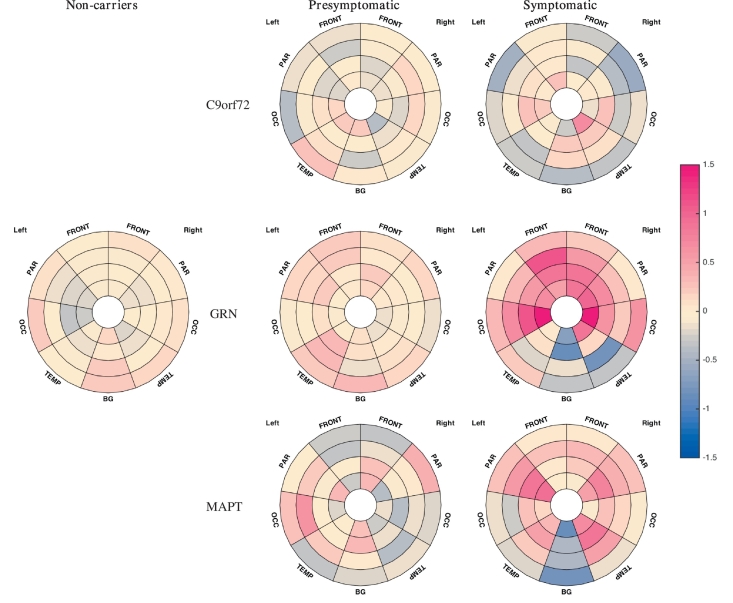
Bullseye representation of the zonal mean of adjusted standardised log-transformed volumes of WMH. Pink reflects a positive Z-score value while blue refers to a negative value.

Beeswarm plots of the raw volumes of lesion and effect size calculated for the mean of log-transformed volumes adjusted for gender, age, TIV, scanner type are presented in Fig. 3 for the four layers and Fig. 4 for the different regions. Significant differences were seen in the symptomatic *GRN* group compared with noncarriers in the three layers nearest the ventricle with no significant differences in the most juxtacortical layer (Fig. 3): layer 1: *p* = 0.0209; layer 2: *p* = 0.0005; layer 3: *p* = 0.0099; layer 4: *p* = 0.3223. Significant differences were also seen in the symptomatic *GRN* carriers compared with the symptomatic *MAPT* and *C9orf72* carriers (layer 1 for *MAPT*, and layers 2, 3 and 4 for *C9orf72*): layer 1: *p* = 0.0457 (*MAPT*), 0.1345 (*C9orf72)*; layer 2: *p* = 0.0539 (*MAPT*), *p* = 0.0147 (*C9or72)*; layer 3: *p* = 0.1314 (*MAPT*), 0.0094 (*C9orf72*); layer 4: *p* = 0.3882 (*MAPT*), *p* = 0.0248 (*C9orf72*). Lastly, significant differences were also seen between the symptomatic and presymptomatic *GRN* carriers for layers 2 and 3: layer 1: *p* = 0.0691; layer 2: *p* = 0.0102; layer 3: *p* = 0.0348; layer 4: *p* = 0.4404. No significant differences were seen between other groups. For the regions, significant differences between the symptomatic *GRN* group and noncarriers were seen in the frontal lobe (*p* = 0.0020) and the occipital lobe (*p* = 0.0072) as well a smaller difference in the parietal region (*p* = 0.0252) with no significant difference observed in the temporal lobe (*p* = 0.6972) (Fig. 4). Significant differences were also observed between the symptomatic *GRN* carriers and the symptomatic *MAPT* and *C9orf72* carriers in the frontal and occipital lobes: frontal: *p* = 0.0170 (*MAPT*), 0.0040 (*C9orf72*); occipital: *p* = 0.0203 (*MAPT*), 0.0196 (*C9orf72*). Furthermore, symptomatic *GRN* carriers had significantly more WMH than presymptomatic carriers in the frontal and occipital lobes: *p* = 0.0184 (frontal), 0.0105 (occipital). Lastly, both *MAPT* and *GRN* symptomatic carriers presented significantly more WMH in the parietal lobe than the symptomatic *C9orf72* carriers: *p* = 0.0117 (*GRN*), 0.0337 (*MAPT*). None of the differences were significant in the temporal lobe. In the basal ganglia, significantly less WMH were detected in the symptomatic *GRN* and *MAPT* cases compared with noncarriers (*p* = 0.0005 (*GRN*), *p* = 0.0002 (*MAPT*)) and with symptomatic *C9orf72* carriers (*p* = 0.0079 (*GRN*), *p* = 0.0055 (*MAPT*)). A symptomatic difference was also seen between the *GRN* symptomatic and presymptomatic carriers (*p* = 0.0016) with other group comparisons nonsignificant. The location analyses are summarized in the bullseye plots in Fig. 5 which encode the mean adjusted standardised value for each zone.

### 3.3 Analysis of lesion appearance

**Fig. 6:**
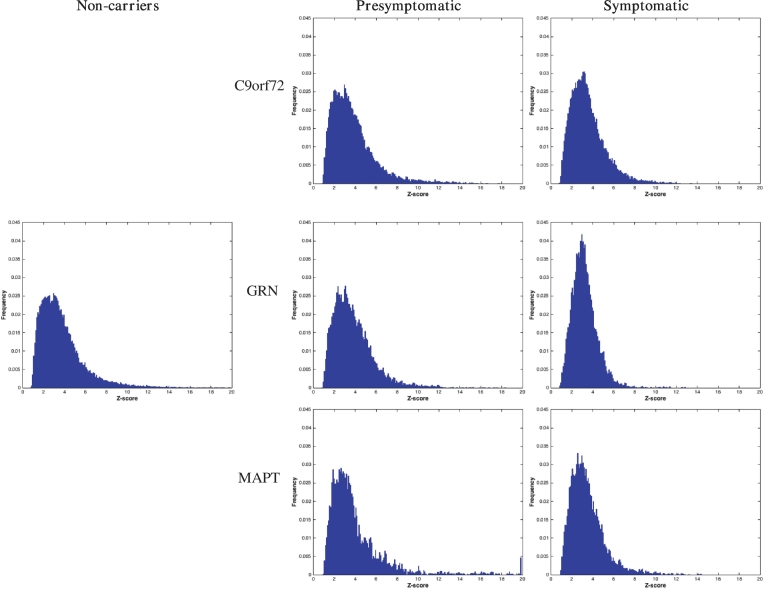
Histogram distribution of T2 level of outlierness for the lesion segmentation in the different groups. Outlierness is measured as a Z-score with respect to the normal appearing white matter.

**Fig. 7:**
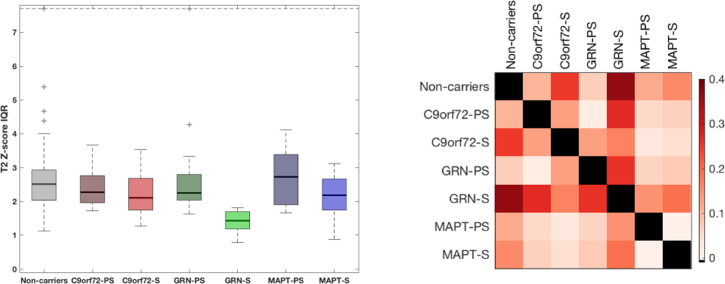
Comparison of inter-quartile range of lesion outlierness in the different groups (left) and corresponding group comparison matrix of effect size.

For the 97 subjects whose WMH volumes were higher than 0.5 mL, histograms of lesion intensities standardised with respect to the normal appearing white matter as obtained by the segmentation model were averaged for each subgroup. Fig. 6 shows the corresponding histogram bar plots. The distribution of lesion intensities in the symptomatic *GRN* group was narrower than for the other groups suggesting greater consistency of signal intensity in the lesions within this group. The interquartile range of the lesion intensity *Z*-scores was used to quantitatively assess this observation: the boxplot of IQR distributions across groups is presented in Fig. 7 along with the effect sizes calculated for the adjusted mean corrected for lesion load and scanner type.

## 4 Discussion

Through the use of a lesion segmentation algorithm adapted to segment WMH from 3D T1- and T2- weighted scans this study extends previous work on WMH in genetic FTD to show their presence in a symptomatic *GRN* mutation group only, and not in presymptomatic participants nor in those with *MAPT* or *C9orf72* mutations. Furthermore, within the *GRN* group there was an association of increased WMH volume with disease progression. Differences were seen in the symptomatic *GRN* group in all three analyses suggesting global and local differences in WMH as well as differences in their appearance.

At the regional level, the frontal lobe was particularly affected by WMH, consistent with previous findings (Paternicò et al., 2016). However, we also found significant differences in the occipital lobe, and to a lesser extent in the parietal lobe. WMH were found in the three most central layers, i.e. closest to the ventricles. It may well be that this periventricular distribution represents a particular pathogenetic feature of *GRN* mutations: progranulin deficiency has been shown to be associated with blood-brain barrier dysfunction and increased permeability (Jackman et al., 2013), which have been shown to be associated with periventricular lesions (Haller et al., 2013). However, as well as the load and location pattern, the intensity outlierness analysis showed that the appearance of the lesions were also different in the symptomatic *GRN* cases from the other groups. The small number of lesions seen in other groups with increasing age are likely to represent small vessel cerebrovascular disease, and therefore the different appearance of the lesions in the *GRN* group may be representative of a non-ischaemic origin. Progranulin has been shown to play a key role in regulating wound repair and inflammation, including affecting tumor necrosis factor alpha signalling (Tang et al., 2011), and progranulin deficiency is known to promote neuroinflammation (Martens et al., 2012): therefore it may be that the lesions seen in these patients are inflammatory in nature. Further evidence for active neuroinflammation in *GRN* carriers comes from studies of knockout *GRN* mouse models which show increased microglial activation (Yin et al., 2010) and increased levels of pro-inflammatory cytokines (Yin et al., 2010), and of blood cytokine levels in human *GRN* mutation carriers which display elevated levels of TNF-α (Miller et al., 2013) and IL-6 (Bossù et al., 2011).

Of note, fewer hyperintensities were observed in the basal ganglia for both the symptomatic *MAPT* and *GRN* groups compared with the other groups. The algorithm cannot differentiate between WMH and enlarged perivascular spaces and in the basal ganglia hyperintensities are likely to correspond to the latter rather than true WMH. It is unclear why fewer are seen in these two groups although this may be related to underlying atrophy of the basal ganglia which tends to be seen in the *GRN* and *MAPT* groups to a greater extent than in the *C9orf72* group (Rohrer et al., 2015). It is less likely to represent a feature of the underlying molecular processes although progranulin is known to promote angiogenesis (Toh et al., 2013) and so progranulin deficiency may potentially be associated with fewer perivascular spaces.

From a technical perspective, the use of this algorithm has advantages over other methods of analysis of WMH location. The application of a patient-specific systematic location scheme prevents the WMH location pattern analysis from suffering from any biases due to registration error or atrophy. Furthermore, the adapted algorithm for T2-weighted images avoids the inclusion of elements at the border between normal tissue that share a common intensity signature with WMH on T2 images. Inherent to the use of T2-weighted images, it must however be noted that in this WMH segmentation, enlarged perivascular spaces are not distinguished from white matter lesions. An additional limitation of the study lies in the fact that no information is given on how individual lesions span multiple zones separated in layers or in lobes. Further investigation of individual lesions both in terms of extent and appearance through texture analysis would be of interest.

The main strength of this study derives from the large cohort available which enables comparisons not only between mutation carriers and non-carriers but also between genetic groups and those at different stages of the disease process. Clinically, the presence of WMH is an important sign of a potential *GRN* mutation in a patient with familial FTD, whilst from a clinical trial point of view, it may be that measurement of WMH load will be a useful biomarker, particularly in trials targeting anti-inflammatory measures. In order to investigate this further, the longitudinal change in load, location and appearance of WMH, and their relationship to other neuroimaging measures of atrophy or neuropsychological measures of disease severity and progression, in *GRN* carriers will be an important subject for future study. Correlation of WMH burden with blood and CSF biomarkers of disease intensity or progression in FTD, or with markers of inflammatory processes, would also be helpful to investigate. In addition, post-mortem studies of patients with *GRN* mutations will also be important to understand what the WMH represent histopathologically, and their relationship, if any, to markers of demyelination, neuronal loss, neuroinflammation, small vessel disease, and TDP-43 pathology.

## Conflicts of interest

None.

## Appendix A Appendix

**List of other GENFI consortium members**

• Christin Andersson - Department of Clinical Neuroscience, Karolinska Institutet, Stockholm, Sweden
• Silvana Archetti – Biotechnology Laboratory, Department of Diagnostics, Civic Hospital of Brescia, Brescia, Italy
• Andrea Arighi - Neurology Unit, Department of Physiopathology and Transplantation, Fondazione Cà Granda, Istituto di Ricovero e Cura a Carattere Scientifico Ospedale Policlinico, Milan, Italy
• Luisa Benussi - Istituto di Ricovero e Cura a Carattere Scientifico Istituto Centro San Giovanni di Dio Fatebenefratelli, Brescia, Italy
• Giuliano Binetti - Istituto di Ricovero e Cura a Carattere Scientifico Istituto Centro San Giovanni di Dio Fatebenefratelli, Brescia, Italy
• Sandra Black - LC Campbell Cognitive Neurology Research Unit, Sunnybrook Research Institute, Toronto, Canada
• Maura Cosseddu - Centre of Brain Aging, University of Brescia, Brescia, Italy
• Marie Fallström - Department of Geriatric Medicine, Karolinska University Hospital, Stockholm, Sweden
• Carlos Ferreira - Instituto Ciências Nucleares Aplicadas à Saúde, Universidade de Coimbra, Coimbra, Portugal
• Chiara Fenoglio - Dept. of Pathophysiology and Transplantation, “Dino Ferrari” Center, University of Milan, Fondazione Cà Granda, IRCCS Ospedale Maggiore Policlinico, Milan, Italy
• Nick Fox – Dementia Research Centre, UCL Institute of Neurology, London, UK
• Morris Freedman - Division of Neurology, Baycrest Centre for Geriatric Care, University of Toronto, Canada
• Giorgio Fumagalli - Neurology Unit, Fondazione Cà Granda, Istituto di Ricovero e Cura a Carattere Scientifico Ospedale Policlinico, Milan, Italy
• Stefano Gazzina - Centre of Brain Aging, Neurology Unit, Department of Clinical and Experimental Sciences, University of Brescia, Brescia, Italy
• Roberta Ghidoni - Istituto di Ricovero e Cura a Carattere Scientifico Istituto Centro San Giovanni di Dio Fatebenefratelli, Brescia, Italy
• Marina Grisoli - Fondazione Istituto di Ricovero e Cura a Carattere Scientifico Istituto Neurologico Carlo Besta, Milano, Italy
• Vesna Jelic - Division of Clinical Geriatrics, Karolinska Institutet, Stockholm, Sweden
• Lize Jiskoot - Department of Neurology, Erasmus Medical Center, Rotterdam, The Netherland
• Ron Keren - University Health Network Memory Clinic, Toronto Western Hospital, Toronto, Canada
• Gemma Lombardi - Department of Neuroscience, Psychology, Drug Research and Child Health, University of Florence, Florence, Italy
• Carolina Maruta - Lisbon Faculty of Medicine, Language Research Laboratory, Lisbon, Portugal
• Lieke Meeter – Department of Neurology, Erasmus Medical Center, Rotterdam, The Netherlands
• Rick van Minkelen - Department of Clinical Genetics, Erasmus Medical Center, Rotterdam, The Netherland
• Benedetta Nacmias - Department of Neuroscience, Psychology, Drug Research and Child Health, University of Florence, Florence, Italy
• Linn Öijerstedt - Division of Neurogeriatrics, Karolinska Institutet, Stockholm, Sweden
• Alessandro Padovani - Neurology Unit, Department of Medical and Experimental Sciences, University of Brescia, Brescia, Italy
• Jessica Panman – Department of Neurology, Erasmus Medical Center, Rotterdam, The Netherland
• Michela Pievani - Istituto di Ricovero e Cura a Carattere Scientifico Istituto Centro San Giovanni di Dio Fatebenefratelli, Brescia, Italy
• Cristina Polito - Department of Clinical Pathophysiology, University of Florence, Florence, Italy
• Enrico Premi - Centre for Ageing Brain and Neurodegenerative Disorders, Neurology Unit, University of Brescia, Brescia, Italy
• Sara Prioni - Fondazione Istituto di Ricovero e Cura a Carattere Scientifico Istituto Neurologico Carlo Besta, Milano, Italy
• Rosa Rademakers - Department of Neurosciences, Mayo Clinic, Jacksonville, Florida
• Veronica Redaelli - Fondazione Istituto di Ricovero e Cura a Carattere Scientifico Istituto Neurologico Carlo Besta, Milano, Italy
• Ekaterina Rogaeva - Tanz Centre for Research in Neurodegenerative Diseases, University of Toronto, Canada
• Giacomina Rossi - Fondazione Istituto di Ricovero e Cura a Carattere Scientifico Istituto Neurologico Carlo Besta, Milano, Italy
• Martin Rossor – Dementia Research Centre, UCL Institute of Neurology, London, UK
• Elio Scarpini - Neurology Unit, Department of Physiopathology and Transplantation, Fondazione Cà Granda, Istituto di Ricovero e Cura a Carattere Scientifico Ospedale Policlinico, Milan, Italy
• David Tang-Wai - University Health Network Memory Clinic, Toronto Western Hospital, Toronto, Canada
• Hakan Thonberg - Center for Alzheimer Research, Division of Neurogeriatrics, Karolinska Institutet, Stockholm, Sweden
• Pietro Tiraboschi - Fondazione Istituto di Ricovero e Cura a Carattere Scientifico Istituto Neurologico Carlo Besta, Milano, Italy
• Ana Verdelho - Department of Neurosciences, Santa Maria Hospital, University of Lisbon, Portugal
• Jason Warren – Dementia Research Centre, UCL Institute of Neurology, London, UK
